# Evaluation of Two Types of Prey on the Developmental Parameters and Population Growth Potentials for a Stinkbug, *Eocanthecona furcellata* via Age-Stage, Two Sex Life Table Method

**DOI:** 10.3390/insects17080834

**Published:** 2026-08-11

**Authors:** Bo Li, Jing Ni, Zhenguo Yang, Yonghong Zhang, Lingli Li, Limin Huang, Xingrong Bai, Yang Hu

**Affiliations:** 1 Sericulture and Apiculture Research Institute, Yunnan Academy of Agricultural Sciences, Mengzi 661101, China; hnlibo02@126.com (B.L.); nixiaoxian@126.com (J.N.); yangzhenguo@yaas.org.cn (Z.Y.); zhang200503@126.com (Y.Z.); lilingli2026@126.com (L.L.); 2 Goodwill Biology (Guangzhou) Co., Ltd., Guangzhou 511470, China; limin.huang@goodwillagro.com; 3 Institute of Plant Protection, Guizhou Academy of Agricultural Sciences, Guiyang 550006, China; 4 Guizhou Key Laboratory of Agricultural Biosecurity, Guiyang 550006, China

**Keywords:** *Eocanthecona furcellata*, *Bombyx mori*, *Tenebrio molitor*, age-stage two sex life table, population performance

## Abstract

*Eocanthecona furcellata* Wolff, a predatory stinkbug, has garnered much attention as a biological control against Lepidoptera, Coleoptera, and Hemiptera pests across various crops. The continuous and high-quality supply of prey is essential for its large scale rearing and commercialization. This study investigated the life history and population performance of *E. furcellata* fed on Lepidoptera prey, *Bombyx mori* larvae or on Coleoptera prey, *Tenebrio molitor* larvae under controlled conditions. Using age-stage two-sex life table methodology, we evaluated key developmental parameters and population growth potentials of *E. furcellata* fed on two types of prey. Results revealed significant differences in pre-adult duration (19.06 vs. 20.47 days), survival rate (0.98 vs. 0.89), adult longevity (27.50 vs. 15.77 days), total pre-oviposition period (25.17 vs. 26.74 days), oviposition days (8.17 vs. 5.37 days), and fecundity (445.54 vs. 222.38 eggs/female) between *B. mori* vs. *T. molitor*-fed population. Population features between *B. mori* vs. *T. molitor*-fed populations, such as the intrinsic rate of increase (0.17 vs. 0.15 d^−1^), finite rate of increase (1.1906 vs. 1.1628 d^−1^), net reproductive rate (218.56 vs. 100.69 ± 22.45 offspring/female), and generation time (3.97 vs. 4.59 days), also diverged markedly. Collectively, *B. mori* larvae were a much better prey than *T. molitor* larvae to rear *E. furcellata*.

## Simple Summary

The predatory stinkbug is a widely used biocontrol agent against many pests in the field. The population performances of the stinkbug could be largely affected by different types of prey. This study evaluated population performances of stinkbugs fed on either domestic silkmoth or mealworm via age-stage, two-sex life table method. The outcomes suggested that the domestic silkmoth was better prey than the mealworm. Stinkbugs fed on domestic silkmoth had a greater survival rate, developmental rate, intrinsic rate of increase, finite rate of increase, net reproductive rate compared to those fed mealworm. Both prey were easy to access, meaning that the food source for rearing this biocontrol agent at large scale would not be a limitation.

## 1. Introduction

*Eocanthecona furcellata* Wolff (Hemiptera: Pentatomidae), an insect predator has been the object of much attention as as biological control against more than 40 insect pests in many crops [1,2,3]. Hemipteran natural enemies, alongside *Orius sauteri* Poppius, *Arma chinensis* Fallou, *Cyrtorhinus lividipennis* Reuter, and so on, play a crucial role in pest management [4]. The developmental stages of *E. furcellata* include egg, five-instar nymph, and adult stages. First-instar nymphs typically lack predatory ability and suck plant sap for about two days before molting into the second instar. From the second to fifth instar, as well as the adult stage, their feeding efficiency increases along with their growth [5]. Known for its strong mobility, high predation efficiency, and broad prey range, this species particularly exhibits biocontrol potentials [6]. Although rearing methods for stinkbugs have been reported to use Lepidopteran insects, such as *Spodoptera frugiperda* Smith, *Plutella xylostella* L., *Bombyx mori* L. (pupa after reeling), *Hyposidra talaca* Moore and one Coleopteran insect, *Tenebrio molitor* L. [3,7,8,9,10], the most efficient rearing method for *E. furcellata* has not been fully established. Furthermore, producing large quantities in an economic way is the basic step to their field use.

The domesticated silkworm, *B. mori* (BM) (Lepidoptera: Bombycidae), has been cultured at a very large scale and in a highly efficiency way over thousand years, meaning it is very easy to obtain a large amount of BM larvae. The body of BM larvae contains approximately 60% protein, 2% fatty acid. The linolenic acid content is 10 times higher than that of milk, and the linoleic acid content is twice that of milk. The total flavonoid content is about 18 mg/g, with trehalose being the primary carbohydrate, along with various vitamins, minerals, lipids, and other nutrients [11]. Thus, BM larvae could be a great food source to rear *E. furcellata* in order to economically produce this predator at large scale. So far, as we know, using BM larvae as the food to rear *E. furcellata* has not been reported. *T. molitor* (TM) (Coleoptera: Tenebrionidae), known as yellow mealworm, has also been produced industrially as food for pets, zoo animals, fishing and even for human consumption [10,12]. TM is considered a good protein source with nutritional value, digestibility, flavor, and several functional abilities. The dried biomass of TM larvae contain approximately 34% protein and 52% fat.

The age-stage two-sex life table is an important method for studying the population growth, survival, reproduction, age differentiation and for developing population prediction models. It has been widely used in population ecology and pest control research [13,14]. The age-stage two-sex life table considers differences in developmental rates between sexes and among individuals. It also describes the relationships of population parameters such as the intrinsic rate of increase *r*, finite rate of increase λ, net reproductive rate *R*_0_, gross reproductive rate *GRR*, and fecundity per female *F* [15]. These methods have been used successfully on many insects [16,17].

Aiming to achieve an economical, continuous, and high-quality prey supply for *E. furcellata* rearing, we used the age-stage, two-sex life table method to compare the key population parameters of *E. furcellata* fed on BM or TM larvae. The outcomes would provide some insights to promote the rearing technique, reducing its rearing costs, and thus enhancing its commercial application in future.

## 2. Materials and Methods

### 2.1. Insects

*E. furcellata* were provided by Goodwill Biology (Guangzhou) Co., Ltd., Guangzhou, China, The *E. furcellata* BM-fed population or TM-fed population were maintained over several generations by feeding on either BM or TM larvae in a laboratory. The BM were provided by the Sericulture and Apiculture Research Institute, Yunnan Academy of Agricultural Sciences, Mengzi, China. The BM larvae were reared on fresh mulberry leaves indoors. The mulberry leaves were collected from the farm maintained by the institute. The TM larvae were purchased from the market and reared on wheat bran before this experiment.

All experimental insects were reared in a temperature-controlled insect-rearing chamber, under standardized environmental conditions: 27 ± 1 °C, 65 ± 10% relative humidity, and a 16 h light/8 h dark photoperiod.

### 2.2. Test Methods

Three egg masses of *E. furcellata* laid on the same day (no less than 40 eggs per egg mass) were collected and kept inside a dry and disinfected plastic box (20 × 13 × 7 cm). Considering the first instar nymphs of *E. furcellata* are unable to prey and feed on a small amount of plant sap, 2–3 fresh mulberry leaves were placed in the box until their first molt. Then, the second instar nymphs that molted on the same day were randomly selected and placed in a box (7 × 7 × 5 cm) individually. There were 53 boxes (replications) set. The box that received TM larva was also placed in a water-soaked sponge (1 × 1 × 1 cm) as water supply since the body of TM larva containing very limited water. In order to provide enough food and keep the box clean, two prey individuals were supplied and replaced daily. Since the body size of early instar of *E. furcellata* is relatively small, 2nd instar larvae of BM were allocated to 2nd instar nymphs of *E. furcellata*, 3rd instar larvae of BM were allocated to 3rd instar nymphs of *E. furcellata*, and 4th instar larvae of BM were allocated to 4th, 5th, instar nymphs and adults of *E. furcellata*, respectively. The TM used to feed *E. furcellata* were all mature larvae. The growth, developmental stage, and mortality of nymphs were recorded daily. The surplus *E. furcellata* nymphs were kept in a dry and disinfected feeding box (35 × 23 × 11 cm) as backup.

The female and male *E. furcellata* adults were counted upon their eclosion. The female and male adults emerged at the same day were paired at a ratio of 1:1. These unpaired adults were discarded. A pair were also kept inside a box (7 × 7 × 5 cm). For the convenience of observing and collecting egg masses, a brown egg laying card was placed inside the box. The survival status of female and male adults, the laid and hatched eggs were recorded on a daily basis. If a male adult died, a new male was introduced until the female died.

### 2.3. Construction and Analysis of Two Sex Life Table of E. furcellata

Twosex-MSChart software (Version 2025.02.18) [18] was used to calculate developmental duration, longevity, adult preoviposition period (*APOP*), total preoviposition period (*TPOP*), fecundity, age-stage specific survival rate (*s_xj_*) (*x* is age, *j* is developmental stage, the same below), age-specific survival rate (*l_x_*), age-stage specific fecundity (*f_xj_*), age-specific fecundity (*m_x_*), age-specific maternity (*l_x_m_x_*), age-stage life expectancy (*e_xj_*), age-stage reproductive value (*v_xj_*), and population parameters including, intrinsic rate of increase (*r*), finite rate of increase (*λ*), net reproductive rate (*R*_0_), and mean generation duration (*T*) [14,15,19,20]. The variance and standard error for all parameters were estimated using the bootstrap technique with 100,000 resamples.

The age-stage specific survival rate (*s_xj_*) refers to the probability of an egg developing to age *x* and stage *j*. Among them, $n_{0,1}$ is the number of individuals used at the beginning of the life table study, and *n_xj_* is the number of individuals who survived to age *x* and stage *j*.

(1) $$S_{xj}=n_{xj}/n_{0,1}$$

The age-specific survival rate (*l_x_*) refers to the probability of a newborn individual surviving until age *x*. *m* is the number of stages.

(2) $$l_{x}=\sum_{j=1}^{m}S_{xj}$$

The age-specific fecundity (*m_x_*) refers to the average number of eggs produced by the entire population at age *x*.

(3) $$m_{x}=\left(\sum_{j=1}^{m}S_{xj}f_{xj}\right)/\sum_{j=1}^{m}S_{xj}$$

The intrinsic rate of increase (*r*) represents the mean daily growth rate of the population as it reaches its stable age-stage distribution.

(4) $$\sum_{x=0}^{\infty }e^{-r\left(x+1\right)}l_{x}m_{x}=1$$

The finite rate of increase (*λ*) refers to the average daily growth rate while the population reaches its stable growth rate.

(5) $$\lambda =e^{r}$$

The net reproductive rate (*R*_0_) refers to the expected offspring number produced by an individual over its entire lifespan.

(6) $$R_{0}=\sum_{x=0}^{\infty }l_{x}m_{x}$$

The mean generation duration (*T*) refers to the time required for a population to increase *R*_0_ time under a stable age-stage distribution.

(7) $$T=\ln R_{0}/r$$

The age-stage life expectancy (*e_xj_*) represents the remaining lifespan for individuals at age *x* and stage *j*. Where *S*′*_iy_* is the probability that an individual of age *x* and stage *j* will survive to age *i* and stage *y*, which is calculated under the assumption that *s_xj_* = 1.

(8) $$e_{xj}=\sum_{i=x}^{\infty }\sum_{y=j}^{m}S_{iy}^{\prime }$$

The age-stage reproductive value (*v_xj_*) refers to individuals at age *x* and stage *j* contributing to the population future.

(9) $$v_{xj}=\frac{e^{r\left(x+1\right)}}{S_{xj}}\sum_{i=x}^{\infty }e^{-r\left(i+1\right)}\sum_{y=j}^{m}S_{iy}^{\prime }f_{iy}$$

#### 2.3.1. Prediction of Population Dynamics of *E. furcellata*

TIMING MSChart software (Version 2025.02.18) [21] was used to project the population dynamics based on age-stage two-sex life table data. The population prediction began with 10 eggs and was projected for 60 days to evaluate their population growth potential and their population characteristics [22].

#### 2.3.2. Data Process

The mean and standard errors of the growth, development, and reproduction parameters of *E. furcellata* were calculated using the Twosex MSChart software [18]. The Paired Bootstrap Test method was applied for significance testing of differences [23]. All relevant charts were drawn using SigmaPlot 15.0 software.

## 3. Results

### 3.1. The Comparisons of Developmental Duration, Reproductive Capacity of E. furcellata

Eleven of 19 recorded parameters of *E. furcellata* nymphs and adults of two populations showed significant differences (Table 1). The developmental duration of egg and the first instar nymph of BM-fed population and TM-fed population were same (7.00 ± 0.00 d). The developmental duration of 2nd, 3rd, and 4th instar nymphs of BM-fed population were shorter significantly than these of TM-fed population. The duration of 5th instar nymphs between populations showed no difference. The preadult developmental duration of BM-fed population was significantly shorter (nearly 1.5 d) than that of the TM-fed population. The pre-adult survival rate *Sa* (0.98 ± 0.02) of BM-fed population (0.89 ± 0.04) was nearly 10 percent higher than TM-fed population. The longevities of female (22.65 ± 2.20 d), male (32.35 ± 3.20 d), and adult (27.50 ± 2.03 d) of BM-fed population were all longer than these of TM-fed population, respectively. In addition, the longevity of females was shorter than that of males, both BM and TM-fed population. The ratio of reproduced females *RepF*/*Fn* of the BM-fed population (0.92 ± 0.05) was 13 percent higher than TM-fed population (0.79 ± 0.08). Compared with the TM-fed population, the BM-fed population had shorter *APOP* and *TPOP*, but longer oviposition days and oviposition period.

### 3.2. Construction of Two-Sex Life Table of E. furcellata

#### 3.2.1. Population Parameters of *E. furcellata*

Most parameters of the BM-fed population were greater than those of the TM-fed population (Table 2). The intrinsic rate of increase (0.1744 ± 0.0061 d^−1^), finite rate of increase λ (1.1906 ± 0.0073 d^−1^), net reproductive rate *R*_0_ (218.5660 ± 42.0188 offspring/female), gross reproductive rate *GRR* (286.4676 ± 56.0102 offspring/female), average egg production per female *F* (445.5385 ± 59.6966 eggs/female), proportion of female to total individuals *Fn*/*N* (0.4906 ± 0.0685), and total longevity (45.9057 ± 2.0883 d) of BM-fed population were all higher than those of the TM-fed population, respectively. The mean generation duration *T* of the BM-fed population (30.8861 ± 0.3559 d) was slightly longer than that of the TM-fed population (30.5770 ± 0.3557 d). The population doubling time *DT* of BM-fed population (3.9741 ± 0.1430 d) was significant shorter than that of the TM-fed population (4.5954 ± 0.2309 d).

#### 3.2.2. The Age-Stage Survival Rates of *E. furcellata*

**Figure 1:**
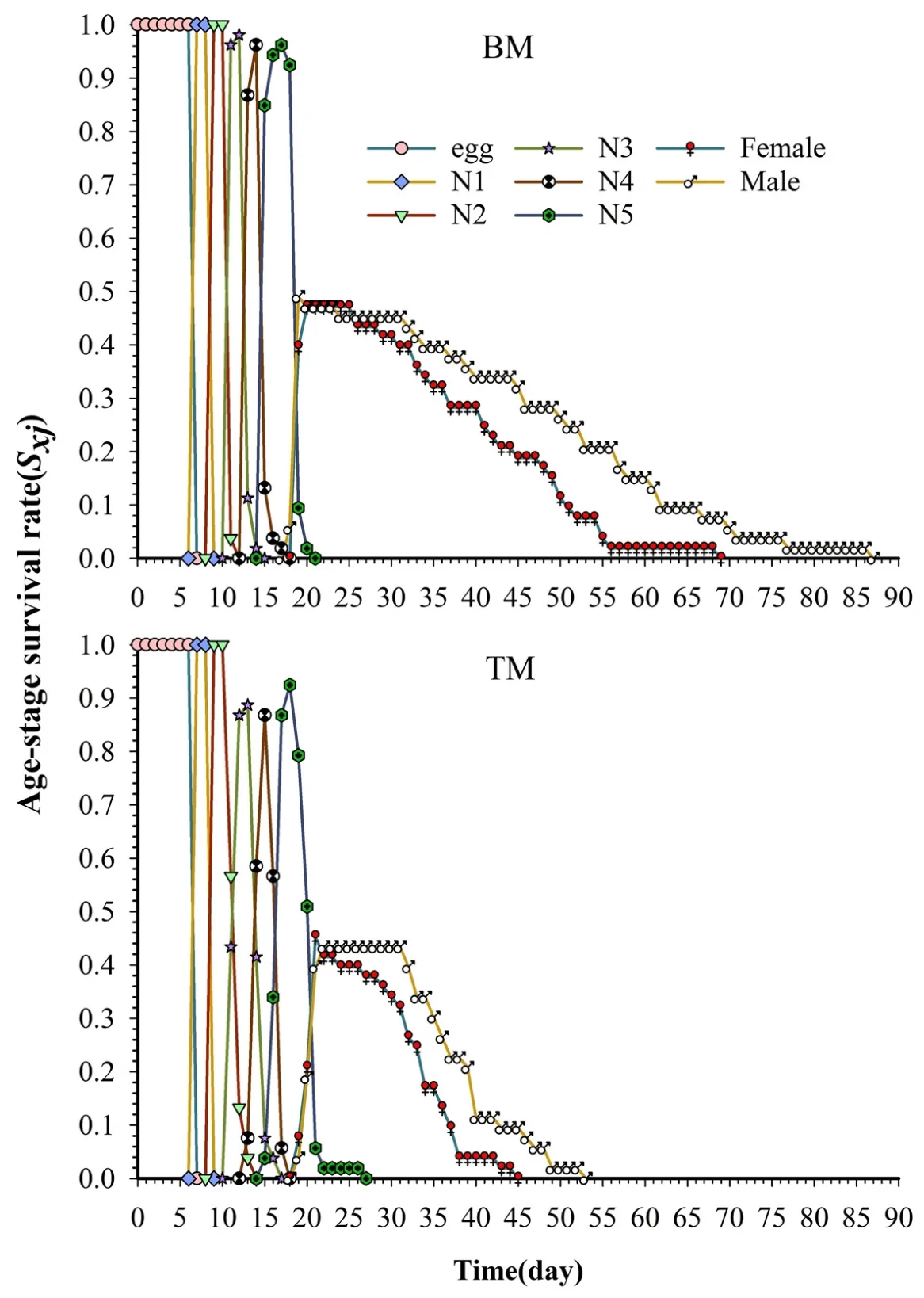
Age-stage survival rate (*S_xj_*) of BM-fed or MT-fed population of *Eocanthecona furcellata*.

The age-stage survival rates *S_xj_* of *E. furcellata* of BM and TM-fed populations were shown in Figure 1. The BM-fed population had a higher survival rate and longer lifespan compared to the TM-fed population. Except for the egg stage, the *S_xj_* of both populations increased at the early age and decreased at the late age. In the adult stage, the *S_xj_* of the BM-fed population declined (all individuals died by 87 d) slower than that of the TM-fed population (all individuals died by 53 d). In addition, the male’s lifespan of both populations were longer than female’s.

#### 3.2.3. The Age-Specific Survival and Fecundity-Related Parameters of *E. furcellata*

**Figure 2:**
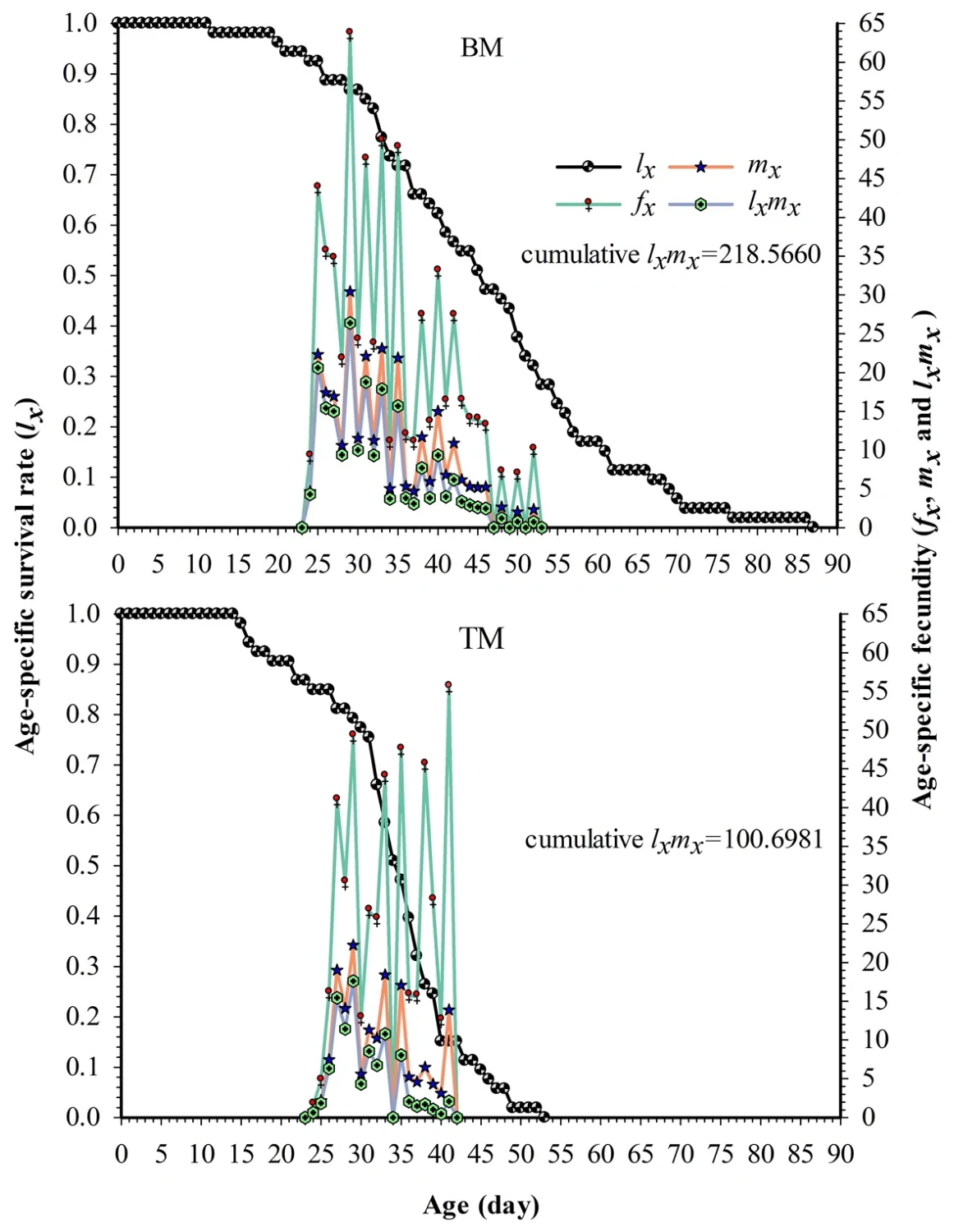
Age-specific survival rate (*l_x_*), fecundity (*f_x_*), age-specifc fecundity (*m_x_*), and age-specific maternity (*l_x_m_x_*) of BM-fed or MT-fed population of *Eocanthecona furcellata*.

As showed in Figure 2, the age-specific survival rates *l_x_* of both populations gradually declined along with increased age. The *l_x_* of BM-fed population declined slower than that of TM-fed population, e.g., all individuals of the BM-fed population were dead at 87 d, while TM-fed population were dead at 53 d, similar to the *S_xj_* in the adult stage (Figure 1). And the cumulative age-specific maternity *l_x_m_x_* (cumu *l_x_m_x_*) was equivalent to the net reproductive rate (*R*_0_). Parameters (*f_x_*, *m_x_,* and *l_x_m_x_*) of the BM-fed population started at 23 d, peaked at 29 d and ended at 53 d, lasting 31 days, with the cumu *l_xmx_* 218.5660. While these three parameters of the TM-fed population also started at 23 d, but *f_x_* peaked at 41 d, *m_x_* and *l_x_m_x_* peaked at 29 d, all ended at 42 d, lasting 20 days only, with the cumu *l_xmx_* 100.6981.

#### 3.2.4. The Age-Stage Reproductive Value V_xj_ of *E. furcellata*

**Figure 3:**
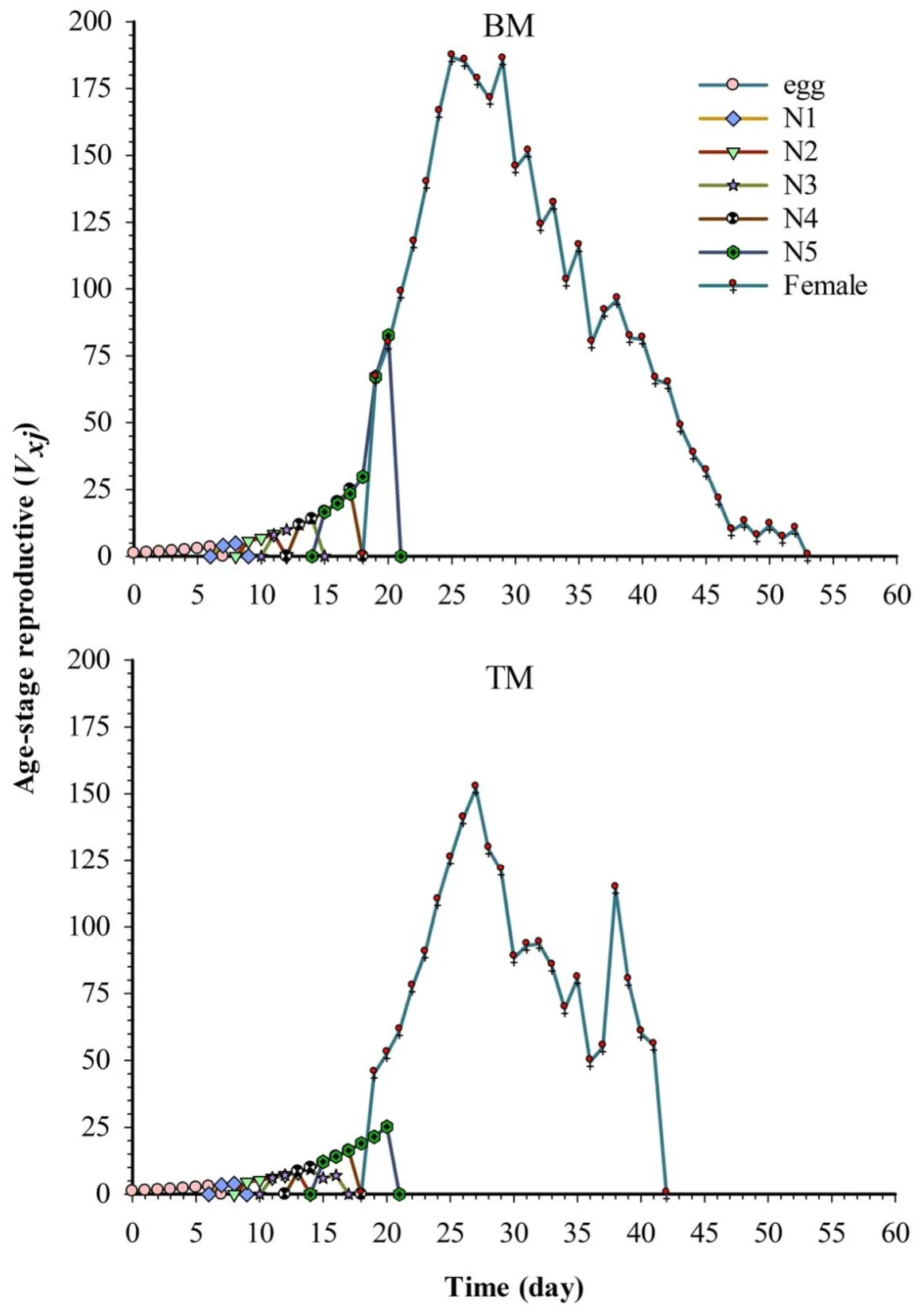
Age-stage reproductive value (*v_xj_*) of BM-fed or MT-fed population of *Eocanthecona furcellata*.

The age-stage reproductive values *v_xj_* of *E. furcellata* of the BM-fed population and TM-fed population are shown in Figure 3. The age-stage reproductive value describes the contribtion of individuals at age *x* to future population growth. The reproductive value at the egg stage *v_xj_* is the finite rate of increase *λ*. The finite rate of increase *λ* in the BM-fed population was 1.19 and 1.16 in the TM-fed population, both were consistent with the finite rate of increase *λ* (Table 2). The values of *λ* were increased gradually during egg, 1~5 instar nymph, but increased quickly after emergence as females. The values of males were 0 in both populations. Overall, the BM-fed population had greater *l_x_*, *f_xj_*, *m_x_*, *l_x_m_x_*, and *v_xj_* compared to the TM-fed population (Figure 2 and Figure 3, Table 1).

#### 3.2.5. The Age-Stage Life Expectancy E_xj_ of *Eocanthecona furcellata*

**Figure 4:**
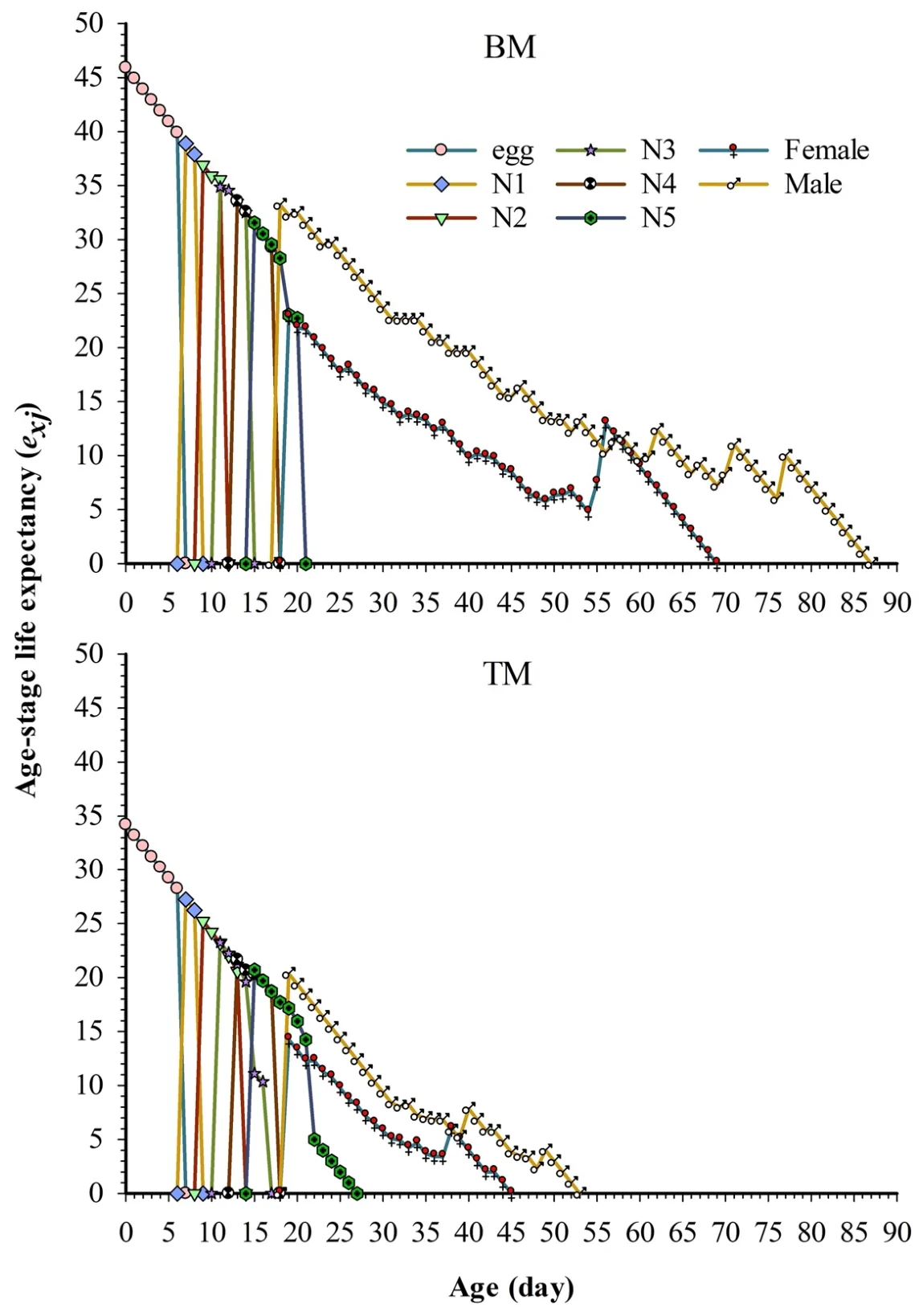
Age-stage life expectancy (*e_xj_*) of BM fed or MT-fed population of *Eocanthecona furcellata*.

The age-stage life expectancy *e_xj_* of all ages in BM and TM-fed populations showed an overall declining trend within a given stage (Figure 4). Notably, the *e_xj_* of fifth-instar nymphs, female adults, and male adults (aged 19–20 and 19–21 d) exhibited significant overlap. The highest life expectancy values of both BM and TM-fed populations occurred at the egg stage (1 d), with the initial values of 45.91 and 34.21, respectively, consistent with total longevity (Table 2). Additionally, the life expectancies of female adults in BM and TM-fed populations were shorter than those of male adults. The maximal life expectancies of female and male adults (69 and 87 d) in the BM-fed population were 24 and 34 days longer than in the TM-fed population (45 and 53 d), respectively.

### 3.3. Population Prediction of E. furcellata

**Figure 5:**
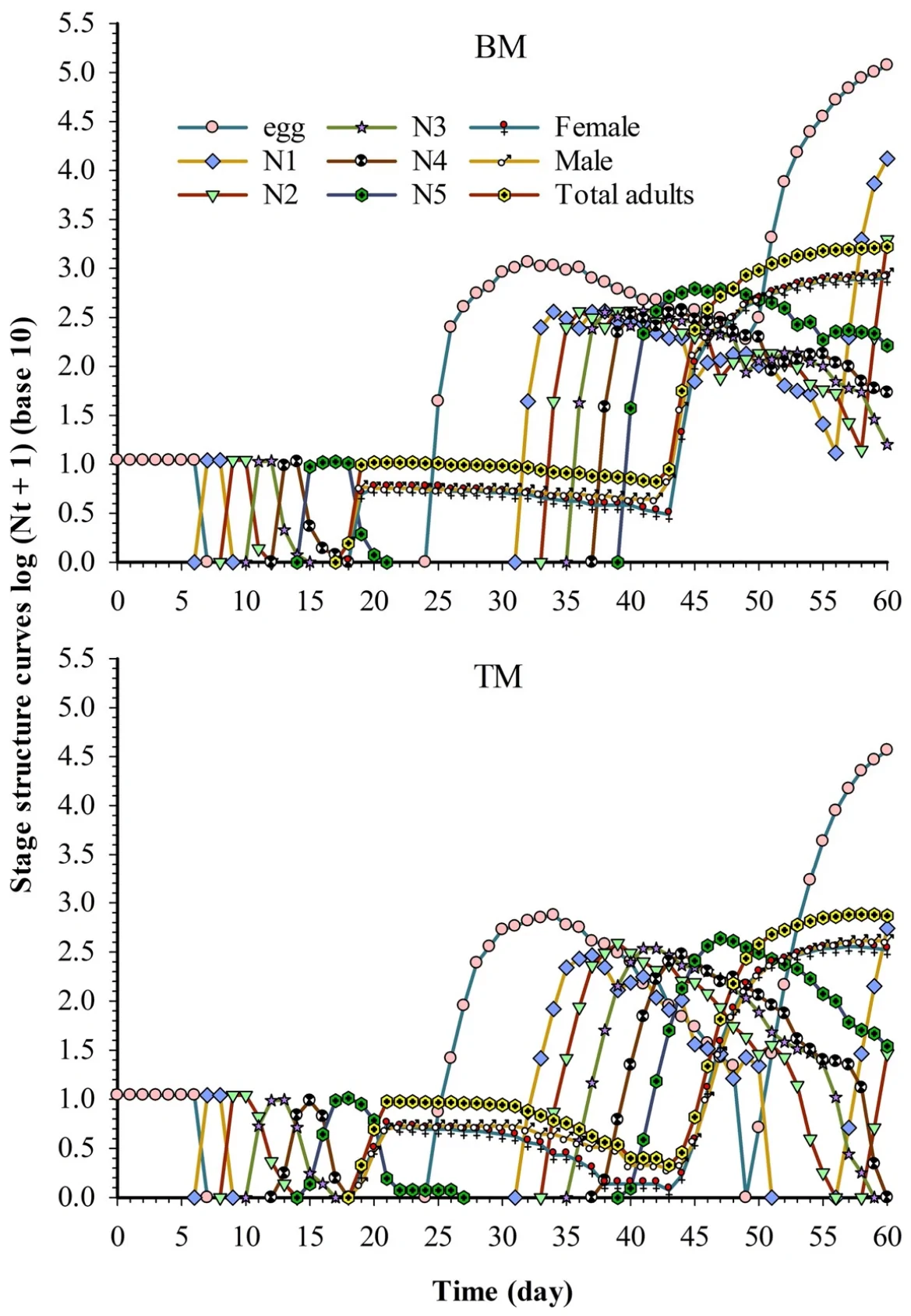
Population prediction of BM fed or MT-fed population of *Eocanthecona furcellata*.

Started with 10 eggs of *E. furcellata*, the predicted population growth and structure over 60 days of BM and TM-fed populations were shown in Figure 5. Both populations could develop into the third generation, but their population structure differed remarkably. By 60 d, the BM-fed population had 135,489.79 individuals (118,385.17 eggs, 13,236.95, 1967.58, 14.91, 52.97, and 162.24 1st to 5th instar nymphs, 802.10 females and 867.80 males), which was 3.55 times that of the TM-fed population 38,210.43 individuals (36,859.14 eggs, 551.09, 28.08, 0, 0, and 33.68 1st to 5th instar nymphs, 330.06 females and 408.38 males). Both BM and TM-fed populations exhibited overlapping of all stages on 40 d.

## 4. Discussion

Developmental parameters are crucial indicators for evaluating food suitability for insects [24,25]. In comparison, the shorter developmental duration, higher survival rate, and greater reproductive capacity of the BM-fed population suggested that BM larvae were better prey than TM larvae. This study constructed an age-stage two-sex life table for the predator *E. furcellata* using BM and TM larvae as prey and described the population life cycle from its beginning (100% survival rate) to its ending (0% survival rate) with parameters such as age-stage survival rate *Sxj* and age-specific survival rates *lx* and so on. The outcomes of the life table also suggested that BM larvae were much better food than TM larvae. To better understand the population structure features and differences of *E. furcellata* feeding on BM or TM larvae, we used a computer program simulating the stage structure and population size within 60 d of *E. furcellata* fed on two types of prey. The results showed that both populations could develop into the third generation within 60 d, but there were significant differences in developmental rates, reproductive parameters, and life table parameters.

Net reproductive rate (offspring/female), a key indicator to estimate the potential of a biocontrol agent, is very sensitive to food sources. Even fed on Lepidopteran larvae, *E. furcellata* varied its net reproductive rates in a great range, as high as 292.4 offspring/female when fed on *Plutella xylostella* larvae [26] and as low as 41.59 when fed on *Spodoptera frugiperda* larvae [27]. In our study, *E. furcellata* had 218.56 offspring/female when fed on BM larvae, close to the upper level of these reports. When fed on TM, a Coleoptera, net reproductive rate in our study was 100.69 offspring/female; about 20 percent higher than the result reported by Yuan et al., 2024 [28]. Intrinsic rate of increase and finite rate of increase have been reported by several studies [3,26,27,28,29], among these reports, both the highest intrinsic rate of increase (0.149) and finite rate of increase (1.161) were reported by Sarkar et al., 2021 when fed on *Hyposidra talaca* larvae [3]. In our study, intrinsic rate of increase and finite rate of increase of BM-fed population were 0.1744 and 1.1906, respectively. Both were greater than those fed on *H. talaca* larvae. These comparisons suggest that BM larvae are a good food for rearing *E. furcellata*.

Based on age-stage sex-specific life table method, the effects of two types of prey on the population fitness of *E. furcellata* were systematically analyzed. The results indicated that BM-fed population significantly outperformed TM-fed population in terms of survival rate, adult longevity, fecundity, net reproductive rate, and finite rate of increase, BM-fed population exhibited higher age-stage specific survival rates and specific age survival rates at all developmental stages compared to the TM-fed population (Figure 1 and Figure 2). In contrast to the BM-fed population, the survival rate of the TM-fed population during the adult stage showed a “cliff-like” decline, suggesting a physiological stress or insufficient nutrient reserves during this stage. BM larvae may provide a more balanced nutritional composition, supporting individuals to maintain physiological functions for a longer period during the adult stage, thereby delaying aging and enhancing survival rate. Life expectancy (Figure 4) reflects the average remaining lifespan of individuals at specific ages and stages. The life expectancy of initiated eggs of the BM-fed population (45.91 days) was significantly higher than that of the TM-fed population (34.21 days), and the life expectancy during the adult stage was also higher, indicating that the BM-fed population had population maintenance capabilities.

Generally speaking, a suitable prey would allow its predator to have a synchronized developmental rate. Thus, developmental uniformity is another key indicator for estimating the quality of prey, which is influenced by various factors such as genetics, temperature, food, density, etc. The greater difference, the longer time required for the entire population to complete development. The more overlapped *S_xj_* curves and greater variances were observed. The variances of 2–5 instar nymphs and preadult developmental duration of BM-fed population were all smaller than those of the TM-fed population (Table 1). The curve of age-stage survival rate *S_xj_* in the TM-fed population was more overlapped than that of the BM-fed population (Figure 1). These results all suggest that BM larvae were better prey than TM larva. In addition, this might be the first empirical data reported about this natural enemy.

Nutrients contained in food are crucial to meeting growth, development, and reproductive needs [30,31]. Nutritional analysis reveals that BM larvae are a nutrient-dense resource. The dry matter contains up to 50–65% crude protein with balanced and abundant amino acids, unsaturated fatty acids (such as linoleic acid), as well as plenty of minerals (iron, zinc) and vitamins [32]. In addition, the protein quality of prey is crucial for the natural enemy’s reproduction. The significantly higher net reproductive rate of the BM-fed population (218.57 offspring/female) vs. the TM-fed population (100.70 offspring/female) suggests that the protein provided by BM larvae had a more balanced amino acid profile.

Unsaturated fatty acids, such as linoleic acid and alpha linolenic acid, are important precursors for the biosynthesis of eicosanoids in insects. Especially, a group of eicosanoids termed prostanoids are involved in reproduction and immune responses. Eicosanoids are key signaling molecules regulating oocyte maturation, ovulation, and egg-laying processes [33]. Azizpor et al. (2024) reported that the exogenous unsaturated fatty acids could directly stimulate the synthesis of eicosanoids in fruit flies and enhance their immune response [34]. More importantly, a single prostaglandin receptor has been identified in fruit flies, which is crucial for insect metamorphosis [35]. In comparison, BM larvae might optimize reproductive outputs by providing fatty acids that are more directly related to reproduction. On the contrary, TM, with higher chitin content, high fat/protein ratio, and low moisture content [36] not only affects the feeding efficiency of predators, but its fatty acid profile may also be unfavorable for the sustained synthesis of reproductive signals.

The sterols are the essential precursors for ecdysteroid production. All sterols of insects must derive from their diet since they can not synthesize sterols de novo [37,38]. The sterols provided by BM, such as cholesterol, are more readily converted into 20-hydroxyecdysone (20E). Evolutionary genomics studies confirmed that Lepidoptera, such as BM, possess a complete sterol conversion enzyme system (e.g., DHCR24). The system is able to convert plant sterols into cholesterol efficiently. Whereas TM’s conversion pathway is more complex and less efficient [39]. Furthermore, the comparative genomic analyses revealed that Coleoptera generally lack the critical conversion enzyme, its homologous gene is absent in all sequenced beetle genomes [39,40], which may affect their sterol nutritional quality as prey. This potential difference in conversion efficiency could reduce 20E titer in TM-fed populations, disrupting the synchronization of development and reproduction, resulting in prolonged pre-adult stages, shortened adult lifespan (27.50 days vs. 15.77 days), and even shortened egg-laying periods of predators.

The uptake of secondary metabolites from plants by herbivores would have a great impact on its predator. Herbivores, for example, BM larvae, may accumulate functional secondary metabolites such as flavonoids from the mulberry leaves. These substances can positively regulate the growth, immune capacity, and reproductive performance of predators through trophic cascade pathways [41]. De Jesus et al. (2014) demonstrated that chemicals (*Cry1Ac* toxin) acquired by a herbivore (fall armyworm) from plants (cotton) could be transmitted along the food chain and significantly influence biological parameters of their natural enemies [42]. The difference lies in the fact that BM transmitted plant-derived beneficial components, while transgenic cotton conveyed insecticidal toxins. These facts form a complete evidence chain of “coexisting positive and negative effects” in the three-level trophic interaction (plant–herbivore–predator). Previous studies have also reported that TM may adversely affect the development and reproduction of stink bugs when TM is the primary food [43,44]. The effects of the above nutrients and secondary metabolites should be investigated piece by piece in further research.

Beside the effects of nutrients and secondary metabolites, physical differences of prey might have great impacts on the growth of natural enemies. Compared to TM, the body of BM larvae contains higher moisture content (70–80% in fresh weight) and softer cuticles, which allow the stinkbug’s stylet piercing through easier and enable faster venom diffusion. The hard chitin epidermis of TM larvae, in comparison to the soft epidermis of BM, might make them much more time-consuming to catch and process as prey. In addition, the lower moisture, higher protein vs. fat ratio of TM larvae might slow down the diffusion of venom produced by *E. furcellata*. In contrast, the bodies of BM larvae were better for the spread of venom because of the higher water content, thus reducing catching and processing time for the predator. Further behavioral studies should also be conducted to improve rearing efficiency.

## 5. Conclusions

In summary, via the age-stage two-sex life table method, we evaluated the growth, development, and reproductive fitness of *E. furcellata* fed on BM larvae and TM larvae. The tested parameters showed that BM larvae were better prey than TM larvae for the large-scale rearing of *E. furcellata*. This study compared the population performance when fed on two types of prey. In the future, which nutritional factor or secondary metabolite plays the most important role should be systematically investigated.

## Figures and Tables

**Table 1:** Developmental duration and fecundity parameters of BM-fed or TM-fed population of *Eocanthecona furcellata*.

Developmental Duration	Population (n)		Mean ± SE		Variance	
	BM Fed	TM Fed	BM Fed	TM Fed	BM Fed	TM Fed
Egg (d)	53	53	7.00 ± 0.00	7.00 ± 0.00	0.0000	0.0000
1st instar nymph (d)	53	53	2.00 ± 0.00	2.00 ± 0.00	0.0000	0.0000
2nd instar nymph (d)	53	53	2.04 ± 0.03 a	2.74 ± 0.11 b	0.0007	0.0115
3rd instar nymph (d)	52	51	2.10 ± 0.04 a	2.63 ± 0.07 b	0.0017	0.0046
4th instar nymph (d)	52	49	2.06 ± 0.03 a	2.29 ± 0.08 b	0.0010	0.0058
5th instar nymph (d)	52	47	3.87 ± 0.05	3.81 ± 0.06	0.0022	0.0042
Preadult duration (d)	52	47	19.06 ± 0.06 a	20.47 ± 0.11 b	0.0040	0.0126
Preadult survival rate *Sa*	52/53	47/53	0.98 ± 0.02 a	0.89 ± 0.04 b	0.0004	0.0019
Female longevity (d)	26	24	22.65 ± 2.20 a	12.88 ± 1.19 b	4.8320	1.4259
Male longevity (d)	26	23	32.35 ± 3.20 a	18.78 ± 1.25 b	10.2118	1.5502
Adult longevity (d)	52	47	27.50 ± 2.03 a	15.77 ± 0.95 b	4.1412	0.9066
Number of female *Fn* (n)	26	24	26.00 ± 3.63	24.00 ± 3.61	13.1945	13.0467
Reproduced female number *RepF* (n)	24	19	24.00 ± 3.61	19.00 ± 3.47	13.0583	12.0712
Ratio of reproduced female *RepF*/*Fn*	24/26	19/24	0.92 ± 0.05	0.79 ± 0.08	0.0028	0.0070
Number of Male *Mn* (n)	26	23	26.00 ± 3.64	23.00 ± 3.61	13.2174	13.0645
Adult pre-ovi period *APOP* (d)	24	19	5.92 ± 0.19	6.37 ± 0.30	0.0354	0.0884
Total pre-ovi period *TPOP* (d)	24	19	25.17 ± 0.18 a	26.74 ± 0.33 b	0.0311	0.1088
Oviposition days (d)	24	19	8.17 ± 0.86 a	5.37 ± 0.59 b	0.7309	0.3519
Oviposition period (d)	24	19	13.83 ± 1.68 a	7.68 ± 0.92 b	2.8158	0.8428

Note: n indicates the number of surviving insects at each stage; the mean, standard error (SE), and variance were estimated through 100,000 paired bootstrap resamplings. Data followed by different letters within the same row indicate significant differences (*p* < 0.05, based on 100,000 paired bootstrap resamplings).

**Table 2:** Population parameters of BM-fed and MT-fed population of *Eocanthecona furcellata*.

Parameters	Population (n)		Mean ± SE		Variance	
	BM Fed	TM Fed	BM Fed	TM Fed	BM Fed	TM Fed
Intrinsic rate of increase *r* (d^−1^)	53	53	0.1744 ± 0.0061 a	0.1508 ± 0.0072 b	0.000037	0.000052
Finite Rate of Increase λ (d^−1^)	53	53	1.1906 ± 0.0073 a	1.16281 ± 0.0084 b	0.000053	0.000071
Net Reproductive Rate *R*_0_ (offspring/female)	53	53	218.5660 ± 42.0188 a	100.6981 ± 22.4575 b	1765.578	504.3411
Mean Generation Duration *T* (d)	53	53	30.8861 ± 0.3559	30.5770 ± 0.3557	0.126686	0.126525
Population Doubling Time *DT* (d)	53	53	3.9741 ± 0.1430 a	4.5954 ± 0.2309 b	0.020448	0.053332
Gross reproduction rate *GRR* (offspring/female)	53	53	286.4676 ± 56.0102	166.0733 ± 48.1494	3137.150	2318.365
Average Eggs Produced per Female *F* (eggs/female)	26	24	445.5385 ± 59.6966 a	222.3750 ± 37.0753 b	3563.688	1374.580
Proportion of Female to Total Individuals *Fn*/*N*	53	53	0.4906 ± 0.0685	0.4528 ± 0.0682	0.004797	0.004602
Total Longevity (d)	53	53	45.9057 ± 2.0883 a	34.2075 ± 1.1637 b	4.360811	1.354192

Note: n indicates the number of surviving insects at each stage; the mean, standard error (SE), and variance were estimated through 100,000 paired bootstrap resamplings. Data followed by different letters within the same row indicate significant differences (*p* < 0.05, based on 100,000 paired bootstrap resamplings).

## Data Availability

The original contributions presented in this study are included in the article. Further inquiries can be directed to the corresponding authors.

## Abbreviations

The following abbreviations are used in this manuscript:

BM	*Bombyx**mori*
TM	*Tenebrio molitor*
N1–N5	1st to 5th instar nymphs of *Eocanthecona furcellata*
